# Clinical and radiological effect of medialized cortical bone trajectory for lumbar pedicle screw fixation in patients with degenerative lumbar spondylolisthesis: study protocol for a randomized controlled trial (mPACT)

**DOI:** 10.1186/s13063-018-2504-z

**Published:** 2018-02-20

**Authors:** Anja Tschugg, Pujan Kavakebi, Sebastian Hartmann, Sara Lener, Christoph Wipplinger, Wolfgang N. Löscher, Sabrina Neururer, Matthias Wildauer, Claudius Thomé

**Affiliations:** 1 Department of Neurosurgery, Medical University of Innsbruck, Anichstr. 35, A-6020 Innsbruck, Austria; 2 Department of Neurology, Medical University of Innsbruck, Innsbruck, Austria; 3 Department of Medical Statistics, Informatics and Health Economics, Medical University of Innsbruck, Innsbruck, Austria; 4 Department of Neuroradiology, Medical University of Innsbruck, Innsbruck, Austria

**Keywords:** Lumbar degenerative disc disease, Transforaminal lumbar interbody fusion, Cortical bone trajectory, Pedicle screw fixation, Minimally invasive technique

## Abstract

**Background:**

Spinal fusion with pedicle screw fixation represents the gold standard for lumbar degenerative disc disease with instability. Although it is an established technique, it is nevertheless an invasive intervention with high complication rates. Therefore, minimally invasive approaches have been developed, the medialized bilateral screw pedicel fixation (mPACT) being one of them. The study objective is to evaluate prospectively the efficacy and safety of the mPACT technique compared with the traditional trajectory for degenerative lumbar spondylolisthesis.

**Methods/design:**

This is a single-center, randomized, controlled, parallel group, superiority trial. A total of 154 adult patients are allocated in a ratio of 1:1. Sample size and power calculation were performed to detect the minimal clinically important difference of 10%, with an expected standard deviation of 20% in the primary outcome parameter, the Oswestry Disability Index, with power of 80%, based on an assumed maximal dropout rate of 20%. Secondary outcome parameters include the EuroQoL 5-Dimension questionnaire, the Beck Depression Inventory, the painDETECT questionnaire and the “timed up and go” test. Furthermore, radiological and health economic outcomes will be evaluated. Follow up is performed until 5 years after surgery. Major inclusion criteria are lumbar degenerative spondylolisthesis with Meyerding grade I or II, which qualifies for decompression and fusion by medialised posterior screw placement with cortical trajectory (mPACT) or by a traditional trajectory for lumbar pedicle screw placement.

**Discussion:**

This trial will contribute to the understanding of the short-term and long-term clinical and radiological postoperative course in patients with lumbar degenerative disc disease, in which the mPACT technique is used.

**Trial registration:**

ISRCTN registry, ISRCTN99263604. Registered on 3 November 2016.

**Electronic supplementary material:**

The online version of this article (10.1186/s13063-018-2504-z) contains supplementary material, which is available to authorized users.

## Background

Spinal fusion with pedicle screw fixation represents the gold standard of surgical treatment of lumbar degenerative disc disease (DDD) with instability of the spinal segment in recent years. Fusion is achieved by creating a firm osseous bridge between the affected levels. Thus, a cage that is inserted via the transforaminal route (transforaminal lumbar interbody fusion (TLIF)) is commonly used to restore the intervertebral disc and foraminal height [1]. Although pedicle screw fixation with transforaminal lumbar interbody fusion is an established technique, it is nevertheless an invasive intervention with high complication rates [2].

Regardless of the popularization of tube-based unilateral minimally invasive (MIS) approaches for TLIF, bilateral posterior exposure was not initially considered possible to be performed in a less invasive fashion. The introduction of the cortical bone trajectory for the placement of medialized bilateral pedicle screw and rod fixation (mPACT) has changed the requirement for wider exposure in posterior fusion and has led to the development of a further MIS posterior approach [3–5]. Although various reports on mPACT have been published recently, there are no randomized, controlled, prospective clinical trials with long-term follow up on this issue. Its application is determined predominantly by the individual surgeon’s preference and skill [3, 6, 7].

The study objective is to prospectively evaluate the efficacy and safety of medialized cortical bone trajectory for lumbar pedicle screw placement compared with the traditional trajectory for degenerative lumbar spondylolisthesis. Both surgical approaches are well-established techniques in clinical practice.

## Methods/design

### Study design

The mPACT study is a single-center, randomized, controlled, parallel group, superiority trial with 154 patients allocated to the groups in a 1:1 ratio. Any trial involving a surgical procedure precludes surgical investigators from being blinded. Patients, however, will be blinded to the randomized treatment and further evaluations, such as radiologic analyses, will be conducted in a blinded fashion whenever possible. The expected enrollment time is 2 years. The conclusion of the study is estimated 5 years thereafter. The primary study endpoint is the difference in Oswestry Disability Index (ODI) between treatment groups at 2 years after surgery. The ODI is a widely used tool for the assessment of therapeutic effect. It consists of ten sections, with six questions in each section. A lower score indicates a higher level of function. An overall score of all 10 sections of the ODI will be computed and used as the ODI score. The standardized version of the ODI can be computed by re-scaling the score to the range 0–100 [8]. Moreover, the generic health status is assessed with the EuroQoL 5-Dimension questionnaire (EQ-5D) [9]. The Beck Depression Inventory (BDI) is used as a multiple choice self-reported inventory for measuring the severity of depression and responsiveness to treatment [10]. To identify neuropathic pain components the painDETECT questionnaire (PD-Q) will be used [11]. Furthermore, with the “timed up and go” (TUG) test the physical ability of walking is objectively documented and quantified [12]. Preoperative and postoperative magnetic resonance imaging (MRI) of the lumbar spine is performed. The image protocols include sagittal and axial sequences, both T1 and T2 weighted, in all patients [13–17]. A computed tomography (CT) scan is routinely performed one year after the intervention to assess the overall fusion rate [18]. Pre-operative American Society of Anesthesiologists (ASA) grade staging might allow the identification of risk factors. Neurological status and the quality and quantity of current pain medication are documented. Operation time and time of hospitalization is recorded. Pain medication usage, including epidural injections and nerve block injections are documented. Quantitative sensory testing (QST) is performed to assess and quantify low back pain non-invasively [19]. Blood samples will be tested preoperatively and postoperatively for TNF-α, IL-6, C-reactive protein (CRP) and leukocytes [20]. Direct costs of hospital care and the indirect costs of follow-up treatment outside the hospital will be evaluated. Costs of surgery and hospitalization including duration of inpatient stay, costs of nursing, costs of medication and physiotherapy are assessed after discharge from the hospital, according to the hospital’s internal cost-estimate lists. Additionally, the indirect costs after hospital discharge including physiotherapy, rehabilitation centers, pain medications, medical consulting etc. are documented on a routing sheet. Postoperative care is not standardized in the study protocol and will be performed according to the standard of care for degenerative lumbar spondylolisthesis at the study site. Primary and secondary outcome parameters are outlined in more detail in Table 1. Ethics approval was attained at the local ethics committee. This study complies with the World Medical Association Declaration of Helsinki Ethical Principles for Medical Research Involving Human Subjects, 2008. The final report will follow the CONSORT 2010 guidelines and its extension to non-pharmacological interventions. The CONSORT checklist is attached as Additional file 1.

**Table 1:** Primary and secondary outcome parameters

Outcome parameters	Assessments
Primary outcome parameter	Oswestry Disability Index (ODI) at 2-year follow up to measure degenerative lumbar spondylolisthesis-related disability [8]
Secondary outcome parameter	Questionnaire - Timed “Up and Go” test (TUG) [12] - Beck depression inventory (BDI) [10] - Changes in physical and mental health captured by the Short Form (SF)-12v2 [9] - Core outcome measure index (COMI) [30, 31], patient-orientated outcome questionnaire including - visual analog scale (VAS) for back and leg pain - patient’s satisfaction - work disability - social disability - Pain relief on 100-mm VAS for back pain and leg pain - PainDetect Questionnaire (PD-Q) [11] - EuroQoL 5-Dimension (EQ-5D) [9]
	Intraoperative - loss of blood (LOB) - amount of blood transfusion - operative time - length of wound incision - type of wound closure
	Various - duration of hospitalization - device-related complications - surgery-related complications - re-operations - adverse events (AE), severe adverse events (SAE)
	Health care contacts
	Pain medication usage
	Hospital costs
	Radiological evaluation - MRI [13–16, 32] - x-ray evaluation (antero-posterior, lateral, flexion and extension, standing for overall sagittal alignment and differences in lordosis) - CT scan (overall fusion rate, rate of radiological and/or symptomatic adjacent segment disease, rate of pedicle and/or cage system implant failures)
	Serum markers (CRP, leukocytes, TNF-α, IL-6)

The primary study endpoint is the difference in Oswestry Disability Index between treatment groups at 2 years after surgery

*MRI* magnetic resonance imaging, *CT* computed tomography, *CRP* C-reactive protein

### Study population

The mPACT trial will include patients with lumbar degenerative spondylolisthesis of Meyerding grade I or II, who qualify for decompression and fusion of ≤ 3 lumbar spinal levels by a posterior medialized cortical bone trajectory or by a traditional trajectory for lumbar pedicle screw placement. These participants need to have failed adequate conservative or interventional therapy for a minimum of 3 months. Additionally, radiologically determined pathologic change at the treatment level needs to correlate with the primary symptoms. Patients with significant morbidities need to be excluded as this may lead to differences in treatment efficacy. Inclusion and exclusion criteria are described in detail in Table 2. Informed consent is obtained from each patient.

**Table 2:** Inclusion and exclusion criteria

	Criteria
Initial inclusion criteria	Age between 18 and 85 years Clinical signs of low back pain and/or radiculopathy from vertebrae L1 to S1 MRI and CT confirmed: central canal stenosis, lateral recesses stenosis, or foraminal stenosis leading to: - radiculopathy, defined as pain and/or motor weakness or paralysis and/or paraesthesia in at least one specific nerve root distribution from vertebrae L1 to S1 or - neurogenic intermittent claudication, defined as pain and/or weakness and/or abnormal sensation in the legs during walking or prolonged standing or - indicating decompressive surgery and instrumented mono-segmental, bi-segmental or tri-segmental spondylodesis with posterior instrumented fusion system and an intervertebral cage (TLIF) Unresponsive to non-operative treatment for a minimum of 3 months including at least physiotherapy, pain medication and local infiltration therapy Presence of progressive symptoms or signs of nerve root and/or spinal cord compression although performing conservative treatment Psychosocial, mental and physical ability to understand and to perform with this protocol, especially visiting scheduled follow-up controls, observe treatment plan and all other study-related procedures Personally signed and dated informed consent document prior to any study-related procedures indicating that the patient has been informed of all pertinent aspects of the trial
Initial exclusion criteria	Previous surgery: (a) any instrumented lumbar spinal surgery, (b) cervical and/or (c) thoracic spinal disease to the extent that surgical consideration is likely or anticipated within 6 months after the lumbar surgical treatment Other degenerative joint diseases (i.e. shoulder, hip knee) to the extent that surgical consideration is likely or anticipated within 6 months after or before the lumbar surgical treatment Any other physical diseases (e.g. neuromuscular disorders) before and/or within 6 months after lumbar surgical intervention, which are able to restrict study procedures (i.e. wheelchair bound) or preclude accurate clinical examination or outcome Severe obesity (BMI >35 kg/m^2^) Neoplasia as the source of symptoms Fixed or permanent neurological deficit unrelated to the lumbar spine disease Active or chronic infection, systemic or local, including HIV, AIDS, hepatitis Active malignancy defined as a history of any invasive malignancy, except non-melanoma skin cancer, unless the patient has been treated with curative intent and there have been no clinical signs or symptoms of the malignancy for a minimum of 5 years Autoimmune disorder that impacts the musculoskeletal system (i.e. lupus, rheumatoid arthritis, ankylosing spondylitis) Acute episode or major mental illness (psychosis, major affective disorder or schizophrenia) Physical symptoms without a diagnosable medical condition to account for the symptoms, which may indicate symptoms of psychological rather than physical origin Recent or current history of substance abuse (drugs, alcohol, narcotics, recreational drugs) Known allergy to titanium, carbon/PEEK and tantalum or intolerance to any device material
Radiological exclusion criteria	Three or more vertebral levels requiring surgical treatment in the lumbar spine Clinically compromised vertebral bodies at the affected level due to current or past trauma, including osteoporotic fractures Spondylolisthesis according to Meyerding grade III or higher
Various exclusion criteria	Patient is currently pursuing personal litigation Pregnancy or the desire to become pregnant in the next year Prisoner or ward of the state Patient has used another investigational drug or device within the last 30 days prior to surgery

*MRI* magnetic resonance imaging, *CT* computed tomography, *BMI* body mass index, *PEEK* Poly-ether-ether-ketone

### Timetable

**Fig. 1:**
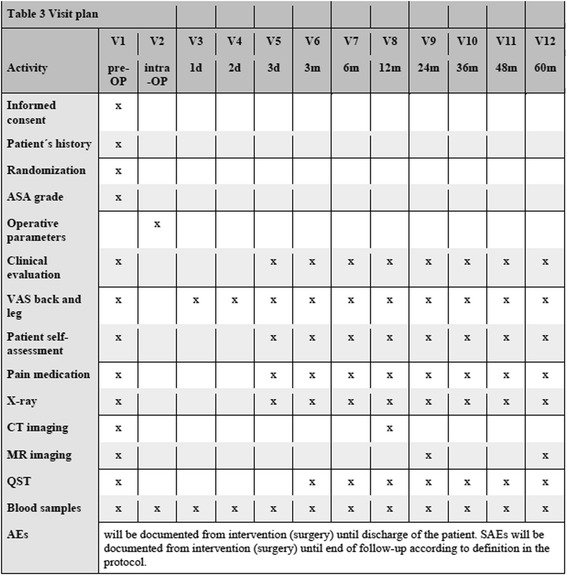
Visit plan. Patients will be followed for 5 years after the intervention. AE, adverse event; ASA, American Society of Anesthesiologists; CT, computed tomography; d, day; intra-OP, intraoperative; m, month; MR imaging, magnetic resonance imaging; QST, quantitative sensory testing; pre-OP, preoperative; SAE, severe adverse event; V, visit; VAS, visual analogue scale

The timetable and visit plan is outlined in Fig. 1.

### Investigational groups

#### Population 1 - titanium-based medialized cortical bone trajectory (mPACT)

**Fig. 2:**
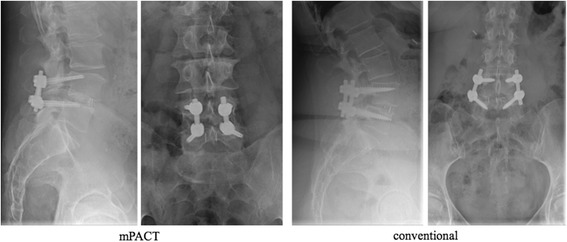
The medialized cortical bone trajectory (mPACT) in comparison to the conventional pedicle srew instrumentation

Cortical bone trajectory, medial-to-lateral, pedicle screws are placed under fluororadiographic guidance. It is performed by identifying the uppermost lateral edge of the pars and moving 3 to 5 mm medial to identify the screw entry point. For the cephalad screw, it is important to avoid the adjacent facet capsule and set the entry point slightly inferior. Typically, the screw is placed with an approximately 15° medial-to-lateral trajectory and an approximately 30° interior-to-superior trajectory for the cephalad screw with a less steep trajectory for the caudal screw, limiting the surgical exposure required [21, 22] (Fig. 2). Decompression is added if necessary. The standard approach to the disc space can be attained via a posterolateral transforaminal (TLIF) route. Then poly-ether-ether-ketone (PEEK) or titan cages filled with bone of the decompression side are used [23, 24].

#### Population 2 - conventional titanium-based instrumentation

The control group needs to represent the gold standard of care and is represented by rigid interbody fusion with titanium-based pedicle screw instrumentation (Fig. 2) [25].

### Randomization

**Fig. 3:**
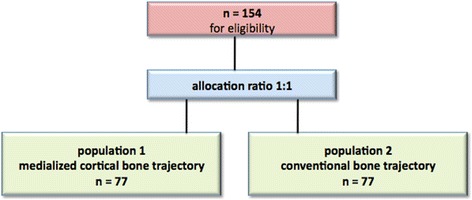
Randomization

Randomization should avoid selection bias for each group. The allocation ratio is 1:1. Allocation of treatments will be performed using a computer-generated list. Statistical Software Stata 10.0 module Ralloc version 3.5.2 (Statacorp College Station, TX, USA) will be used to generate the random code (CODE: ralloc block size treat, ratio(1) osiz(2) nsubj(77) trtlab(mPACT konventionell) strata(2) seed(20161012) sav(*File-Path*) idvar(study_ID)). An independent statistician at the Department of Medical Statistics, Informatics and Health Economics, Innsbruck Medical University will administer the randomization code (Fig. 3).

### Selection bias

Selection bias should be avoided by the randomization of the allocated intervention. All patients accomplishing the inclusion criteria are considered for participation and pre-screening lists are kept to justify non-included patients. Performance bias should be avoided by a standardized protocol of postoperative care at our department. Both groups receive the same preopeative and postoperative treatment until discharge. Attrition bias should be avoided by the “intention-to-treat” or “per-protocol” analysis, respectively. Every patient included in the trial will be considered in the overall evaluation.

### Data management and confidentiality

Study data generation at the study sites is clearly separated from data storage, processing and statistical analysis in the Department of Medical Statistics, Informatics and Health Economics. This requires a validated database system programmed in a customized software package, which is provided at the same place. The system includes an audit trial facility and will be used for defining the database structure, data entry, for handling data cleaning processes, and for final storing of the data. The evaluation of the data takes place by double-entry of the data and manual/visual evaluation of plausibility. The database will be closed at the completion of the study after entry of all collected data and clarification of all queries. All study findings and documents will be regarded as confidential. The investigator and members of the research team must not disclose any information without prior written approval from the Sponsor. The pseudonymity of patients participating must be maintained. Throughout documentation and evaluation, the patients will be recognized on case report forms (CRFs) and other documents by age and identification number. Documents that identify the patient personally (e.g., the signed informed consent form) must be maintained in confidence by the investigator. The patients will be told that all study findings will be stored on computer and handled in strictest confidence.

### Sample size and power calculation

The sample size calculation is based on the primary endpoint of the study, a difference in the ODI 2 years post intervention [8]. The sample size was calculated based on the two-group *t* test (GraphPad StatMate Software, version 2.0). Based on a minimally clinical important change of 10% on the ODI, standard deviation for the ODI of 20%, significance level of *p* ≤ 0.05, and power of 80%, an estimated 64 patients are required in each of the intervention groups, for the primary endpoint. The current sample size of 154 patients allows for a 20% loss to follow up while maintaining > 80% statistical power [23].

### Statistical analysis

Primary and secondary variables and their change from baseline will be summarized by treatment group. Endpoints will be analyzed as appropriate in dependence on the data distribution at a two-sided 0.05 level of significance. Detailed descriptive statistics will be provided for the data collected and 95% confidence intervals will be calculated for all relevant estimates. Measurements on the course of follow up will be analyzed by analysis of covariance (ANCOVA) or generalized model alternatives for categorical or semi-quantitative data. Additional covariates, such as baseline measurements, will be included in the model as appropriate. MRI parameters and variables to assess functional neurological status will be summarized descriptively. Imputation of missing data has not been planned. The primary analysis will follow as randomized. All values will be expressed as the mean and standard deviation.

Data are analyzed for normality by the Kolmogorov-Smirnov test. The Wilcoxon signed-rank test or the paired *t* test is used to analyze continuous paired data, while McNemar’s test is used for categorical data. The Mann-Whitney U test and unpaired *t* test, are used to perform group comparisons of continuous clinical outcome variables while the chi-square and Fisher’s exact test are used to analyze intergroup differences in categorized outcome variables and demographic characteristics (age, sex ratio, duration of symptoms, etc.), respectively. Statistical analysis will be performed after recruitment, one year postoperatively and after completion of the last visit of the study population at the specified time points. An interim analysis will be performed 6 and 12 months after recruitment and after completion of the last visit of the study population at the specified time points.

## Discussion

The high prevalence of spinal disease, especially the increasing number of patients with degenerative disc disease, extensively increases the demand for spinal fusion surgery [26]. The standard surgical technique used is pedicle screw fixation with transforaminal lumbar interbody fusion [1]. Although it is an established technique, it is nevertheless an invasive intervention with high complication rates that appear to increase with age, blood loss, operative time, and the number of levels treated [2]. Therefore, minimally invasive techniques have been developed to minimize tissue damage, reduce blood loss, decrease narcotic requirement, and shorten the length of hospital stay [27]. The mPACT technique was established as one of these techniques. A posterior lumbar interbody fusion (PLIF) is then additionally used to perform an intervertebral fusion [6]. However, the PLIF is associated with higher rates of nerve tissue damage and extends the duration of operative procedure in comparison with TLIF [28]. In our prospective, clinical, randomized, controlled trial we will thus use TLIF only.

Implant loosening, especially in patients with poor bone nutrition, is another mentionable drawback that may be associated with spinal instrumentation [29]. The mPACT technique offers greater cortical bone contact [3] and therefore shows several promising advantages in biomechanical testing in comparison to traditional pedicle screw fixation. The cortical bone trajectory increases the pullout strength and improves the rigidity of spinal instrumentation [4].

Most clinical data on this subject are based on retrospective investigations or small prospective cohort studies without long-term results [3, 6, 7]. At this time, there are no prospective, clinical, randomized, controlled trials available. With current standards, well-validated outcome instruments are mandatory to provide efficient surgical treatment recommendations for patients with lumbar degenerative disc disease. This trial will contribute to the understanding of the short-term and long-term clinical and radiological postoperative course in patients with lumbar degenerative disc disease in which the mPACT technique is used.

### Trial status

The trial will start in January 2017 at the Department of Neurosurgery, Medical University of Innsbruck.

## Additional file

Additional file 1: SPIRIT 2013 Checklist: recommended items to address in a clinical trial protocol and related documents. (DOC 122 kb)

## Abbreviations

AE: Adverse event
BDI: Beck Depression Inventory
BMI: Body mass index
CRP: C-reactive protein
CT: Computed tomography
EQ-5D: EuroQoL-5 Dimension
IL: Interleukin
MIS: Minimally invasive surgery
mPACT: Medialised posterior screw placement with cortical trajectory
MRI: Magnetic resonance imaging
ODI: Oswestry Disability Index
PD-Q: painDETECT questionnaire
PEEK: Poly-ether-ether-ketone
PLIF: Posterior lumbar interbody fusion
QST: Quantitative sensory testing
SAE: Severe adverse event
TLIF: Transforaminal lumbar interbody fusion
TNF: Tumor necrosis factor
TUG: Timed up and go test
VAS: Visual analog scale
